# Characterization and genome sequence of *Microbacterium foliorum* phage Morrigan, of cluster EA6 with siphovirus morphology

**DOI:** 10.1128/MRA.00719-23

**Published:** 2023-11-17

**Authors:** Jennifer A. Guerrero, Nery Maldonado, Emmanuella O. Mbajiofor, Lisette Ramirez, Mary Jo Valencia, Creehan D. Healy, Alejandro A. Garza, Samantha A. Mizell, Brianna N. Jackson, Mayra Vargas Ayala

**Affiliations:** 1 Department of Integrative Biology, University of Texas at San Antonio, San Antonio, Texas, USA; Portland State University, Portland, Oregon, USA

**Keywords:** Bacteriophage, actinobacteriophage

## Abstract

Bacteriophage Morrigan, which was isolated from soil using *Microbacterium foliorum* NRRL B-24224, is lytic with siphovirus morphology. Morrigan’s 40,509-bp genome has a GC content of 62.8% and 66 putative protein-coding genes, of which 31 could be assigned putative functions. Based on gene content similarity to actinobacteriophages, Morrigan is assigned to subcluster EA6.

## ANNOUNCEMENT

**Fig 1:**
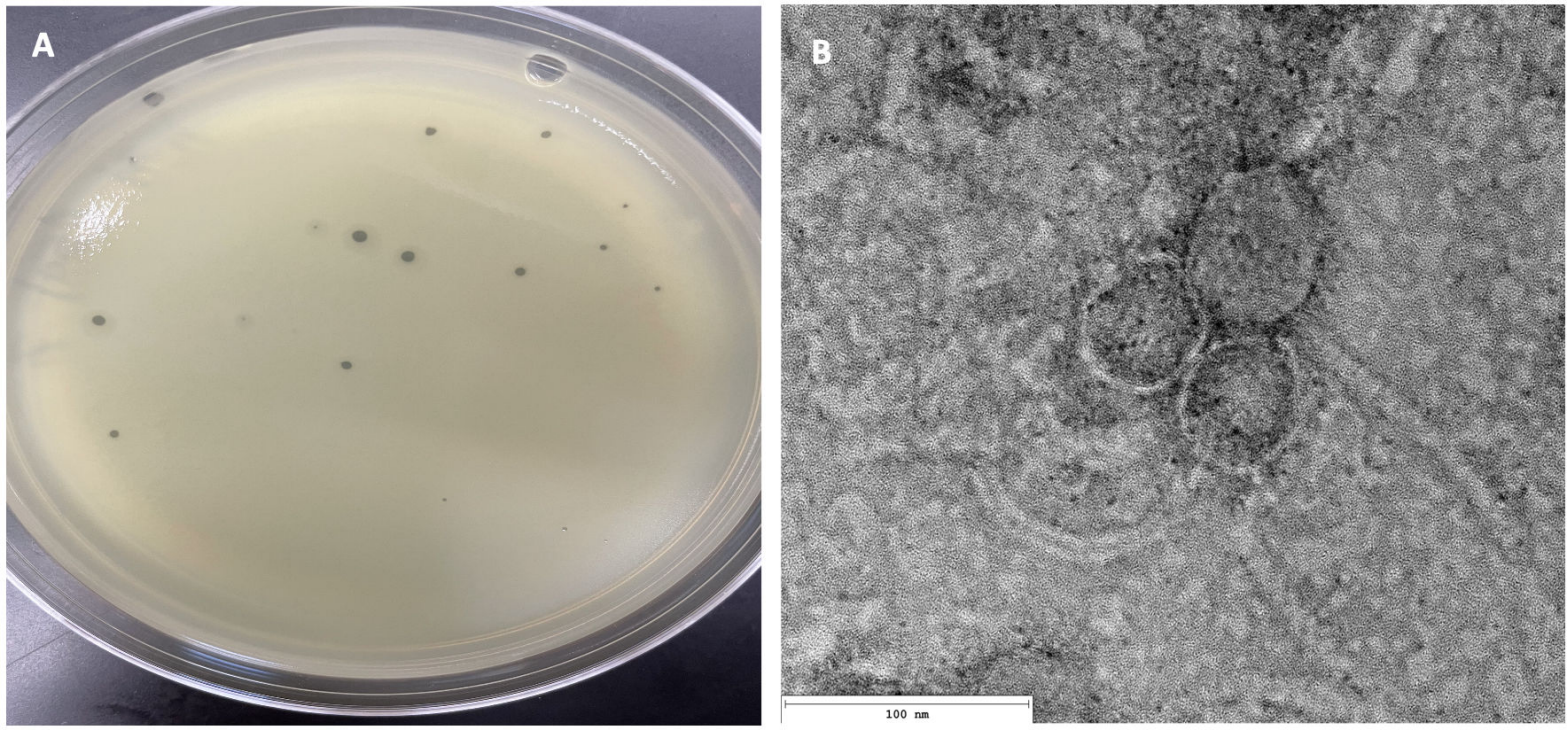
(A) Morrigan produced clear plaques with a clear center and a turbid halo, 1–3 mm (*n* = 4) in size. (**B**) Negative-stain transmission electron microscopy identified Morrigan to have a long flexible tail, 124.0 nm, and an isometric capsid diameter of 56.0 nm (*n* = 1) with siphovirus morphology.

Identification and characterization of novel bacteriophages continue to advance our understanding of viral diversity and evolution as well as serve as a promising avenue for developing strategies for controlling antibiotic-resistant bacterial infections (1–3). Here, we describe actinobacteriophage Morrigan isolated, on 10 July 2022, from topsoil a few centimeter deep near tall grass, San Antonio, Texas (29.58126 N; 98.61998 W) using standard procedures (4). Peptone-yeast extract-calcium liquid media were added to the sample and then incubated at 30°C while shaking for 2 h at 220 rpm. The supernatant was filtered through a 0.22 µm filter and then inoculated with *Microbacterium foliorum* NRRL B-24224. After shaking at 30°C for 2 days, the culture was centrifuged, and the supernatant was plated in a soft agar overlay with *M. foliorum*. Morrigan formed clear plaques with a turbid halo 1–3 mm in diameter after 48 h at 30°C (Fig. 1A) and was purified through two rounds of plating, and negative-staining (1% uranyl acetate) transmission electron microscopy revealed a siphovirus morphology (Fig. 1B).

Viral genomic DNA was extracted from lysate using the Promega Wizard DNA cleanup kit and sequenced. Sequencing libraries were prepared using the NEB Ultra II Library kit and sequenced by Illumina MiSeq sequencer (v3 reagents) yielding 470,386 single-end 150-bp reads, an approximate shotgun coverage of 1,660. Untrimmed reads were assembled using Newbler v2.9 and checked for completeness and genomic termini using Consed v29 (5). The double-stranded DNA genome of Morrigan is 40,509 bp long, circularly permuted, and has a GC content of 62.8%. Based on gene content similarity (GCS) of at least 35% to phages in the actinobacteriophage database (phgaesDB), using the phagesDB GCS tool, Morrigan is assigned cluster EA (subcluster EA6) (6–8).

Morrigan was auto-annotated using DNA Master v5.23.6 (http://cobamide2.bio.pitt.edu) embedded with Glimmer v3.02 (9) and GenMark v2.5p (10). Gene calls were refined using Phamerator (11) and Starterator (v.462; http://phages.wustl.edu/starterator/), while gene functions were assigned using HHpred [using PDB_mmCIF70, SCOPe70, Pfam-A, and NCBI_Concerved_Domains databases (5 May 2023)] (12), BLAST [using NCBI nonredundant and actinobacteriophage databases (5 May 2023)] (13), PhagesDB (6), TMHMM v2.0 (14), and SOSUI v1.11 (15). ARAGORN v1.2.38 (16) and tRNAscan-SE v2.0 (17) were used to search for tRNAs. All programs were run using default settings and following SEA-PHAGES Bioinformatics Guide (18). Morrigan is predicted to contain 66 protein-coding genes, of which 31 could be assigned putative functions. No tRNAs were identified. The genome architecture of Morrigan is similar to phages within sub-clusters EA1–EA8 where one strand transcribes genes on one half and the other strand transcribes genes on the other half of the genome (19). One half of the genome contains genes predicted to encode the major capsid protein, major tail protein, tail assembly chaperone, and endolysin. The other half of the genome contains genes predicted to encode DNA polymerase I, RecA-like DNA recombinase, nuclease, and AAA-ATPase. Like other EA phages, no integrase or repressor functions were identified, suggesting that Morrigan is an obligate lytic phage. Morrigan is currently most significantly aligned with *Microbacterium* phages: Chepli, Luna18, and KatChan with 87.33% nucleotide identity (13).

## Data Availability

Morrigan is available at GenBank with accession no. OR253914 and Sequence Read Archive (SRA) no. SRX19690845.

## References

[1] Russell DA, Garlena RA, Hatfull GF. 2019. Complete genome sequence of Microbacterium foliorum NRRL B-24224, a host for bacteriophage discovery. Microbiol Resour Announc 8:e01467-18. doi:10.1128/MRA.01467-18 30714032 PMC6357638

[2] Kim H-J, Lee AW, Park C. 2018. Toxicological evaluation of Microbacterium foliorum SYG27B-MF. Regul Toxicol Pharmacol 100:16–24. doi:10.1016/j.yrtph.2018.09.022 30308225

[3] Gneiding K, Frodl R, Funke G. 2008. Identities of Microbacterium spp. encountered in human clinical specimens. J Clin Microbiol 46:3646–3652. doi:10.1128/JCM.01202-08 18799696 PMC2576590

[4] Poxleitner, M., Pope, W., Jacobs-Sera, D., Sivanathan, V., & Hatfull, G. (2018). Phage discovery guide. Howard Hughes Medical Institute, Chevy Chase, MD.

[5] Russell DA. 2018. Sequencing, assembling, and finishing complete bacteriophage genomes. Methods Mol Biol 1681:109–125. doi:10.1007/978-1-4939-7343-9_9 29134591

[6] Russell DA, Hatfull GF. 2017. PhagesDB: the actinobacteriophage database. Bioinformatics 33:784–786. doi:10.1093/bioinformatics/btw711 28365761 PMC5860397

[7] Pope WM, Garlena RA, Guerrero-Bustamante CA, Jacobs-Sera D. 2017. Science education alliance-Phage hunters advancing Genomics and Evolutionalry science (SEA-PHAGES)

[8] Pope WH, Mavrich TN, Garlena RA, Guerrero-Bustamante CA, Jacobs-Sera D, Montgomery MT, Russell DA, Warner MH, Hatfull GF. 2017. Bacteriophages of Gordonia spp. display a spectrum of diversity and genetic relationships. mBio 8:e01069-17. doi:10.1128/mBio.01069-17 28811342 PMC5559632

[9] Delcher AL, Bratke KA, Powers EC, Salzberg SL. 2007. Identifying bacterial genes and endosymbiont DNA with glimmer. Bioinformatics 23:673–679. doi:10.1093/bioinformatics/btm009 17237039 PMC2387122

[10] Besemer J, Borodovsky M. 2005. Genemark: web software for gene finding in prokaryotes, eukaryotes and viruses. Nucleic Acids Res 33:W451–4. doi:10.1093/nar/gki487 15980510 PMC1160247

[11] Cresawn SG, Bogel M, Day N, Jacobs-Sera D, Hendrix RW, Hatfull GF. 2011. Phamerator: a bioinformatic tool for comparative bacteriophage genomics. BMC Bioinformatics 12:395. doi:10.1186/1471-2105-12-395 21991981 PMC3233612

[12] Söding J, Biegert A, Lupas AN. 2005. The HHpred interactive server for protein homology detection and structure prediction. Nucleic Acids Res 33:W244–8. doi:10.1093/nar/gki408 15980461 PMC1160169

[13] Altschul SF, Gish W, Miller W, Myers EW, Lipman DJ. 1990. Basic local alignment search tool. J Mol Biol 215:403–410. doi:10.1016/S0022-2836(05)80360-2 2231712

[14] Krogh A, Larsson B, von Heijne G, Sonnhammer EL. 2001. Predicting transmembrane protein topology with a hidden Markov model: application to complete genomes. J Mol Biol 305:567–580. doi:10.1006/jmbi.2000.4315 11152613

[15] Hirokawa T, Boon-Chieng S, Mitaku S. 1998. SOSUI: classification and secondary structure prediction system for membrane proteins. Bioinformatics 14:378–379. doi:10.1093/bioinformatics/14.4.378 9632836

[16] Laslett D, Canback B. 2004. ARAGORN, a program to detect tRNA genes and tmRNA genes in nucleotide sequences. Nucleic Acids Res 32:11–16. doi:10.1093/nar/gkh152 14704338 PMC373265

[17] Lowe TM, Eddy SR. 1997. tRNAscan-SE: a program for improved detection of transfer RNA genes in genomic sequence. Nucleic Acids Res 25:955–964. doi:10.1093/nar/25.5.955 9023104 PMC146525

[18] Pope, W., Jacobs-Sera, D., Russel, A., Cresaw, G., & Hatfull, G. (2017). SEA-PHAGES bioinformatics guide. Howard Hughes Medical Institute, Chevy Chase, MD.

[19] Jacobs-Sera D, Abad LA, Alvey RM, Anders KR, Aull HG, Bhalla SS, Blumer LS, Bollivar DW, Bonilla JA, Butela KA, et al.. 2020. Genomic diversity of bacteriophages infecting Microbacterium spp. PLoS One 15:e0234636. doi:10.1371/journal.pone.0234636 32555720 PMC7302621

